# Summary of the proceedings of the International Summit 2015: General and subspecialty radiology

**DOI:** 10.1007/s13244-015-0453-6

**Published:** 2016-01-11

**Authors:** European Society of Radiology (ESR)

**Affiliations:** Neutorgasse 9/2, 1010 Vienna, Austria

**Keywords:** General radiologist, Subspecialisation, Multispecialty radiologist, Fragmentation in radiology, Integrity (of radiology)

## Abstract

**Abstract:**

The need for subspecialisation in radiology and the relationship of general and subspecialist radiologists is very diverse in different regions of the world according to the reports presented at the ESR International Summit, organised by the ESR during the European Congress of Radiology in March 2015 in Vienna. The International Summit is held once a year by the ESR and its national and international radiological partner societies from outside Europe with the aim to address and discuss selected subjects of global relevance in radiology. In 2015, the relationship between general and subspecialist radiologists was analysed. It was shown that the situation differs immensely between developed and developing countries; in developed countries, a considerable proportion of radiologists are subspecialty trained; subspecialty radiologists practise mainly in large and academic departments, and many radiologists practise as multispecialty radiologists. In many developing countries only general radiologists—if available at all—practise radiology, and imaging interpretation is often performed by physicians with very limited relevant training or in some cases even by non-physicians.

***Main messages*:**

• *Subspecialisation and preservation of the integrity of the radiology profession are relevant for improved patient care.*

• *Subspecialisation is needed in large departments, providing the basis for innovation and research.*

• *Subspecialty sections should preferably remain within the overarching radiology department.*

• *Shared facilities, efficient use of resources and common organisational structures are beneficial.*

• *A multispecialty radiologist model is an option to build robust academic and private practices.*

## Introduction

The International Summit was established by the European Society of Radiology (ESR) in order to intensify the collaboration of national and international radiological societies from outside Europe and to discuss selected subjects of global relevance in radiology at each European Congress of Radiology (ECR). So far, at the ECR 2013 and ECR 2014 the relationship between radiology and nuclear medicine, and the position of ultrasound in radiology, were discussed, while the topic of “General radiologist versus subspecialist radiologist” was discussed at ECR 2015. Representatives of the following radiological societies, usually the president and one or two members of the executive, were invited to this meeting to present the situation in their respective country or region: American College of Radiology (ACR), Asian Oceanian Society of Radiology (AOSR), Canadian Association of Radiology (CAR), Inter-American College of Radiology (CIR), Colombian Association of Radiology (CAR), International Society of Radiology (ISR), Japan Radiological Society (JRS), Korean Society of Radiology (KSR), Radiological Society of North America (RSNA), and the European Society of Radiology (ESR). Representatives of several past “ESR meets” countries/societies were also invited to attend the meeting: Brazilian College of Radiology, Egyptian Society of Radiology and Nuclear Medicine, Radiological Society of South Africa and Mexican Radiology and Imaging Society.

It was demonstrated that radiology practice differs among different regions of the world in many aspects, and that the need for subspecialisation in radiology and the relationship between general and subspecialist radiologists are very diverse. The most pertinent comments presented by the participants of the International Summit at the ECR 2015 and the discussions and conclusions about the relationship of general versus subspecialist radiologists in different parts of the world are presented in this paper.

## Situation in Europe

The relationship of general and subspecialist radiologists in Europe is complex and very diverse regarding the number of radiologists and organisational issues between countries and hospitals. The largest academic departments in many small central European countries have a maximum number of 35 radiologists and most hospitals have departments with only 10–15 practising radiologists. The academic hospitals in large, highly developed European countries have a much higher number of radiologists and can organise subspecialist radiology services much more easily. The added value of the modern radiologist to the patient is primarily to communicate with clinicians and advise on imaging, to relate images and reporting, and to safeguard quality and patient safety. Clinicians have become subspecialised, with an increasing amount of knowledge about an often small part of medicine, and the only way for radiologists to assert their role in such an environment is to be clinical partners, with equal knowledge about a medical subspecialty and to take an active part in clinical decision-making. Thus, a clinical model is advocated where radiologists should communicate on image interpretation, not only by report/PACS but also in multidisciplinary team and direct patient discussions.

Initially, subspecialisation occurred based on different modalities in the 1970s and 1980s, while later organ-system based subspecialisation became much more appropriate and was gradually adopted both in the structure of radiological services and in the training curricula.

Radiologists are dependent on referrals from others and many imaging modalities are very attractive for other professions; other physicians tend to provide their own imaging services in their respective subspecialty clinical areas and turf battles are a reality for radiologists worldwide. Fragmentation or break-away of some parts of the imaging services from the radiology department and erosion of the radiological domain is a real challenge. Fragmentation has negative effects on the profession and services provided to patients since it separates those outside the imaging speciality from advances in the general field, prevents them from cooperating with other radiologists and makes them too one-sided and thus less valuable to patients [1].

Quality and safety standards of radiological services are crucial. There are many advantages of preserving the integrity of radiology as a speciality, such as appropriate patient referral, skill in image interpretation and image-guided interventions, broad clinical perspective, technology mastery, recognition of technical artefacts, dose reduction techniques, standardised workflow, quality and safety issues, 24/7 services and clustering of equipment. Another important issue is also the avoidance of self-referral if physicians refer patients to radiologists.

The ESR position, as stated by B. Brkljačić and C.M. Owens, is that a well-balanced approach is needed to satisfy both the clear need for subspecialisation in radiology, and at the same time to preserve the integrity of the profession and avoid its fragmentation. Fifteen ESR Subspecialties and Allied Sciences Societies (institutional member societies of the ESR) are much stronger if closely aligned within rather than outside the ESR. The ESR European Training Curriculum for Radiology (ETC) is a very important contribution to preserving the unity of radiology on the European level from the beginning [2]. The ETC (level I-II), version 2014, was supported by 66 ESR National Societies (European and non-European) and all 15 ESR Subspecialties and Allied Sciences Societies. The newest version released at ECR 2015 also comprises a curriculum for a full subspecialisation in a field of radiology corresponding to Level III training, which should be a formal, full-immersion training in a radiological subspecialty with an expected minimum of 1 year after the completion of radiology (Level I and Level II) training. The contents have been provided by the respective ESR Subspecialties and Allied Sciences Societies. In addition, the ESR published a curriculum for undergraduate radiological education. The ETC is a living document and will be revised at regular intervals. All three ESR curricula are available at www.myESR.org/TrainingCurriculum.

## Situation in North America

With regard to radiology training in the United States, nearly all graduating residents do subspecialty fellowships and there is an increasing trend towards training subspecialists. According to Bibb Allen of the American College of Radiology (ACR), in reality nearly all United States private practice radiologists practise in more than one subspecialty area [3]. Statistics show that practices are planning to hire mostly general radiologists over the next 3 years. The ACR paper, “General Radiologist in the 21st century”, presented a new model of multispecialty radiologists as an exciting and viable option to help build robust future academic and private radiology practices, which was, however, not well received by general radiologists [4, 5].

N. Reed Dunnick of the Radiological Society of North America (RSNA) stated that the field of radiology has expanded dramatically and no-one can master everything. Even “general radiologists” now restrict their practice domains to some extent. Furthermore, the subspecialties themselves are often further subdivided, such as neuroradiology into, for example, brain, head and neck spine, paediatrics and interventional neuroradiology. Electronic communication enables referring physicians to refer patients directly to subspecialty radiologists. General radiologists are needed in small and rural places, over nights and weekends, but nevertheless, the question arises whether a general radiologist could do neuroimaging more effectively than a non-radiology neuroscientist, and whether there are actually two levels of care, e.g. at nights or weekends.

According to statistics of radiologists hired in the United States in 2013, general radiology leads with 18.2 %. The statistics for the plan of hiring radiologists in the future show that general radiology will be in fourth place with 10 %. Radiologists must better understand the clinical context of examinations and procedures, interact more directly with patients, conduct imaging research, be true experts in their field and thus subspecialise, Dunnick emphasised.

The Future Scanning and Signposts Working Group of the Canadian Association of Radiologists (CAR), chaired by D. Koff under the authority of J. Lévesque (CAR President), have reviewed the role of the general radiologist and its impact on future practice by looking at the definition and three possible scenarios for the future of the general radiologist, namely: maintaining the status quo, evolving into a subspecialised system and evolving into a hybrid system [6]. The general radiologist did not pursue any additional subspecialty fellowship training, while subspecialists pursued additional training in one or more subspecialty areas and can be either exclusive subspecialists or multispecialty radiologists. J. Lévesque stated that the definition of general radiologist would include those radiologists who practised multimodality, multisystem radiology, irrespective of their training. He enumerated the advantages and disadvantages of the three possible scenarios.

Advantages of maintaining the status quo are, among others, that it may well work for some of the larger multispecialty radiologist groups that have in essence already evolved into the hybrid system. Disadvantages are that it is difficult to be competent in all areas unless doing a reasonable volume of examinations. In addition, turf battles and competition with teleradiology firms will increase. Also, there is the potential for subspecialty groups to become disenfranchised and splinter off into new societies, form new billing groups within the same hospital, or integrate into other clinical services with the loss of training capability for future residents.

With regard to the evolution into a subspecialised system, the advantages are a proactive shift to more subspecialisation by current general radiologists through retraining, mini-fellowships, CME and mentorship programs will be seen. Clinicians would be happy with subspecialty reads, resulting in fewer turf wars. Subspecialists are essential in large groups and in academic centres, providing the basis for innovation and research. Disadvantages are that the differentiation in subspecialty requires a critical mass only available in major academic centres and smaller sites want to retain an on-site professional as part of the community. Also, it becomes more difficult to train new residents who are able or wish to work in small communities as general radiologists.

As regards the third possible scenario, i.e. the evolution into a hybrid system, the advantage is that in larger centres, “general” radiologist practice is already evolving; most radiologists have fellowships and they practise multispecialty radiology, except in very large centres. Current general radiologists adapt by either a combination of moving to a smaller community, reading in areas which may be more conducive to a general radiologist, referring some cases for subspecialised interpretations either in-house or outsourced, or by obtaining additional training of 1 year or longer to become subspecialised or multispecialised. Canadian academic centres introduce more multispecialty fellowships, where a fellow can concentrate on three or four areas. Disadvantages include that teleradiology groups with 100 % subspecialty reads still compete for contracts, the volume may need to be defined to maintain competency, and the call is an issue if the area being reported is not read regularly.

Many Canadian radiologists support the concept of multispecialty radiology and agree that the general radiologist is ideally a multispecialty radiologist, he concluded.

## Situation in Latin America

A survey completed by ten Latin American member societies of the Colegio Interamericano de Radiología (CIR) shows a great variability among countries in technology, workforce, training programmes, quality assurance, radiation protection, and research and certification programmes, but also considerable differences within each country with regard to urban versus rural, high versus low income and private versus public healthcare. Resources are concentrated primarily in large urban centres. In 70 % of the countries, there is no regulation of the number of specialists. General radiology is the most frequent practice and very few centres are organised by subspecialties. There is generally a considerable lack of radiologists and subspecialty programmes in Latin America. The programmes are concentrated mainly in five countries: Argentina, Brazil, Chile, Colombia and Mexico. In addition, there is no legal recognition of subspecialties and thus no stimulus for respective training. There is demand for all subspecialties in Latin America, as CIR representative M.R. Casale Menier emphasised.

F.G. Lubinus of the Colombian Association of Radiology (ACR) reported that 17 Colombian universities offer formal and certified training in radiology and diagnostic imaging in seven cities in Colombia, but only 23 % of these universities offer also subspecialty training in six areas of radiology. In Colombia, 78 % of the radiologists practise in five cities, and approximately 85 % of the subspecialised radiologists working in Colombia received their title abroad as there a few places for fellowships in Colombia. Thirty-one percent of the radiologists have no certified training in an area or specialty, but focus on a certain subspecialty by personal interest, experience and self-education. Most of the residents graduating as general radiologists feel the need for additional training in a specific area either through formal training or self-education. Teleradiology is becoming a threat for general radiologists working in provinces since it is considered a less expensive way to provide services by specialised radiologists.

## Situation in Japan and Korea

Among 7,144 diagnostic radiologists, 2,334 (33 %) are members of one of the eight existing subspecialty radiological societies in Japan. However, only 11 % of all diagnostic radiologists and 16 % of all board-certified diagnostic radiologists practise as subspecialist radiologists. H. Honda of the Japan Radiological Society (JRS) stated that subspecialist radiologists work almost exclusively in large university hospitals (13 % of Japanese hospitals), while radiologists working in small hospitals (60 % of Japanese hospitals) are exclusively general radiologists. Radiologists working in medium-sized hospitals (27 % of hospitals) mostly work as general radiologists, and only some of them work in subspecialty areas. Expanding subspecialty areas in Japan are: neuro, chest, cardiac, abdomen and breast.

The Korean Society of Radiology (KSR) counts 13 affiliated subspecialty societies, three of whom are related to interventional radiology. Among 3,311 practising radiologists in Korea, 1,583 are general radiologists, while 1,728 are subspecialist radiologists. Korea currently has 80 training institutions and 613 residents. With regard to the future, KSR representative S.H. Kim claimed that balancing between general and subspecialist radiologists, between KSR and subspecialty societies, between subspecialty societies and counterpart clinical societies, and between academic and non-academic positions is of utmost importance as laid down in the KSR’s new slogan: “Open, Balanced, Sustainable”.

## Situation in Asia and Oceania

K.P. Reddy of the Asian Oceanian Society of Radiology (AOSR) stated that the situation in their member states is extremely diverse with regard to the total number of radiologists, the number of general and subspecialty radiologists, radiology training institutions and number of residents. Due to the extreme differences, it is impossible to generalise the situation in this vast region.

However, in the majority of countries, general radiology is practised the most. Also, most of these countries have an insufficient number of radiologists. Reddy emphasised the need to increase the exchange of fellowship programmes among Asian countries and between the AOSR and international societies as subspecialisation is required for radiology to survive and would be the appropriate response to the general demand. It is important to practise to the same standards as the clinical subspecialists and to keep the referring clinicians convinced that subspecialty competence in radiology is essential.

## Situation in developing countries—position of the International Society of Radiology

J.P. Borgstede of the International Society of Radiology (ISR) reported that in developing countries, imaging interpretation is predominantly performed by individuals with limited training who may not even be physicians. There are largely general radiologists, if radiologists at all and teleradiology is used for subspecialty readings.

Borgstede raised the issue of training in developing countries, i.e. whether only radiologists as defined by the European or United States model, other physicians or other health care providers should be trained. He stated that it may depend on the diseases that can be treated. “If radiologists become a commodity, it will be a global one, which could be bought, sold and traded internationally”, Borgstede said.

## Discussion

Radiologists in Canada are contracted to be physically present in the community and to go to smaller practices, often thousands of kilometres away, and the single-payer model works well. In the United States, it is also very common to send subspecialty radiologists to smaller practices, within 100 miles, to perform tasks such as diagnostic mammography. In many cases, non-radiologists, such as academic radiographers, operate radiology devices in the USA; family practices are trained to interpret studies and mid-level advanced practitioners to read reports. Even nurses try to receive permission to order, interpret and bill the reports.

With an increasing amount of new knowledge and the expansion of radiology it is challenging for the individual radiologist to maintain the necessary expertise in all fields. Radiologists should understand the clinical context of examinations and procedures, interact directly with patients, conduct imaging research, and thus subspecialise, particularly in large academic and community hospitals.

There was consensus that more subspecialisation training is needed, and that subspecialisation is a clear need for radiologists in large academic and community hospitals, and in most cases subspecialty sections should remain within the overarching department of radiology, with the benefit of shared facilities, efficient use of resources and common organisational structures. It seems that many radiologists support the concept of multispecialty radiology and agree that the general radiologist is ideally a multispecialty radiologist. Implementation of a quality control process is mandatory in the near future and all radiologists should comply.

Fragmentation and erosion of the radiological domain have negative effects on the profession and services provided to patients. Knowledge about why, when, where, how and for whom imaging and image-guided interventions should be performed is an integral part of radiology training, ensuring quality and safety standards of radiological services. It is well known that physicians practising self-referral request 4–4.5 times more examinations than physicians referring patients to radiologists.

## Conclusions

The European or United States model of physician training is in place in almost all developed countries. A considerable proportion of radiologists are fellowship or subspecialty trained and many practise as multispecialty radiologists. On the contrary, in many developing countries, imaging interpretation is performed by individuals with limited training who may not even be physicians. In these regions, general radiologists are dominant, if radiologists exist at all. Well-trained general radiologists are needed in order to read simple imaging studies and perform basic procedures and thus cover the demand of imaging in rural areas.

Subspecialisation is a clear need for radiologists in large academic centres, providing the basis for best patient care, innovation and research. A model of multispecialty radiologists is a viable option to build robust academic and private radiology practices, and general radiologists are in many instances evolving to multispecialty radiologists. Implementation of a quality control process is mandatory in the near future and all radiologists should comply.

In order to satisfy the need of subspecialisation in radiology and to maintain the integrity of the profession, which has many advantages, and thus avoid its fragmentation, a well-balanced approach is needed. The advantages of maintaining the integrity are referral, knowledge in image interpretation and image-guided interventions, broad clinical perspective, technology mastery, standardised workflow, quality and safety issues, 24/7 services and clustering of equipment.
